# 6G—Enabling the New Smart City: A Survey

**DOI:** 10.3390/s23177528

**Published:** 2023-08-30

**Authors:** Maurizio Murroni, Matteo Anedda, Mauro Fadda, Pietro Ruiu, Vlad Popescu, Corneliu Zaharia, Daniele Giusto

**Affiliations:** 1 Department of Electrical and Electronic Engineering, University of Cagliari, 09123 Cagliari, Italy; m.murroni@ieee.org (M.M.); matteo.anedda@unica.it (M.A.); ddgiusto@unica.it (D.G.); 2 Department of Biomedical Sciences, University of Sassari, 07100 Sassari, Italy; mfadda1@uniss.it (M.F.); pruiu@uniss.it (P.R.); 3 Department of Electronics and Computers, Transilvania University of Brașov, 500068 Brașov, Romania; vlad.popescu@unitbv.ro

**Keywords:** smart city, smart mobility, 5G, 6G

## Abstract

Smart cities and 6G are technological areas that have the potential to transform the way we live and work in the years to come. Until this transformation comes into place, there is the need, underlined by research and market studies, for a critical reassessment of the entire wireless communication sector for smart cities, which should include the IoT infrastructure, economic factors that could improve their adoption rate, and strategies that enable smart city operations. Therefore, from a technical point of view, a series of stringent issues, such as interoperability, data privacy, security, the digital divide, and implementation issues have to be addressed. Notably, to concentrate the scrutiny on smart cities and the forthcoming influence of 6G, the groundwork laid by the current 5G, with its multifaceted role and inherent limitations within the domain of smart cities, is embraced as a foundational standpoint. This examination culminates in a panoramic exposition, extending beyond the mere delineation of the 6G standard toward the unveiling of the extensive gamut of potential applications that this emergent standard promises to introduce to the smart cities arena. This paper provides an update on the SC ecosystem around the novel paradigm of 6G, aggregating a series of enabling technologies accompanied by the descriptions of their roles and specific employment schemes.

## 1. Introduction

The sixth-generation (6G) wireless communication is the successor of fifth-generation (5G) communication. From a technological point of view, it makes use of higher frequency radio bands and provides a higher capacity combined with lower latency, enabling the integration into a single network with increased throughput and reliability. These characteristics make 6G networks ideal for the large-scale adoption of the Internet of Things (IoT) paradigm, especially considering that, in a short time, the devices connected to the IoT infrastructure are expected to reach billions of devices [1]. At the moment, little information is available on the new 6G wireless standard, but considering the rapid advancements in wireless systems, the arrival of 6G communication is inevitable [2]. The passage to the new 6G standard is expected to be a potential game-changer, as underlined in ref. [3], due to the wider frequency range and enhanced transmission rates, and unlike 5G, 6G does not require high-power consumption. In terms of security, data processing, threat detection, and data encryption are among the issues for which 6G networks will bring improvements by using decentralized security systems and handling the data traffic locally in a dynamic manner [4].

In ref. [5], emerging applications, together with key enabling technologies, are analyzed, outlining current research projects and discussing technical challenges on future research directions toward 6G. Ref. [6] provides a comprehensive portrayal of the 6G vision, technical requirements, and application scenarios also describing some existing testbeds and advanced 6G verification platforms. In ref. [7], one of the key innovations, which is the use of the terahertz (THz) band, is deeply analyzed in terms of channel propagation characteristics, modeling techniques, and measurement capabilities for 6G communication applications, presenting recommendations to allow for efficient use of it. Ref. [8] discusses the roles of 6G, considering five main IoT application domains: healthcare, unmanned aerial vehicles, vehicular and autonomous driving, satellite, and industrial IoT.

In this article, we further explore the main key technologies of 6G, identifying how they can be exploited by another paradigm, which has emerged in the realm of new technologies, i.e., the smart city (SC). An SC is a municipality where communication networks and traditional services are made more efficient through the use of digital and real-time solutions, with technical, economic, and social benefits to residents and businesses. SCs employ digital technologies for better use of resources by ensuring smarter urban transportation networks, better monitoring and water supply facilities, optimized waste disposal, and more efficient ways of lighting and heating buildings. On the other hand, the city government becomes more interactive and responsive, ensuring safer public spaces and meeting the needs of an aging population.

As research and market studies show [2], there is a need for a critical reassessment of the entire wireless communication sector for SCs in the light of energy-efficient communication. This should also include the IoT infrastructure, economic factors that could improve their adoption rate, and strategies that enable their operations of SCs. The development of the SC concept relies heavily on ubiquitous connectivity because the existing communication infrastructure cannot keep pace with the rapid growth and extended operation of SCs, this leading to the main reason for adopting a technology with an increased, energy-efficient transmission rate such as the ones provided by the 6G communication. In conclusion, it is safe to affirm that the development of the SC concept will happen in parallel to the development of 6G technologies [2].

The idea behind this article is to update an SC ecosystem around the novel paradigm of 6G, aggregating the enabling technologies accompanied by a description of their role and employment schemes. Upon a rigorous survey of existing literature, it is evident that while diverse surveys on the subject of 6G are extant, a dearth of sufficiently comprehensive endeavors exists, expounding on the holistic spectrum of 6G’s application potential within SCs. This paper presents a comprehensive survey of 6G and the related technologies in the SC context along with the challenges posed by these emerging paradigms and technologies.

The paper is structured as follows. Section 2 delves into the pivotal enabling technologies that underpin the functioning of SCs. It investigates the critical components and infrastructural elements that drive the realization of SCs, establishing a foundational understanding for subsequent discussions. In Section 3, the paper conducts a concise yet informative analysis of the evolutionary trajectory from preceding mobile network generations to the nascent 6G era. This examination not only traces the historical development but also underscores the significant shifts that have transpired over time. Section 4 is dedicated to a thorough exploration of the resource management intricacies within the realm of SCs, particularly within the context of the 5G era. It navigates through the challenges and complexities that emerge while conceptualizing and implementing SCs against the backdrop of the 5G technological landscape. Section 5 constitutes a detailed narrative, tracing the very genesis and emergence of the groundbreaking 6G technology. This section embarks on a journey that unveils the compelling factors necessitating the development of this next-generation technology, offering an insightful perspective into its motivations. In Section 6, the paper succinctly encapsulates the vast potentialities that 6G technology brings to the realm of SCs. This segment delineates how the integration of 6G can catalyze innovative solutions and amplify the capabilities of SCs, fostering a holistic and transformative urban ecosystem. Section 7 elucidates a panoramic overview of SCs empowered by 6G technology, drawing insights from an array of pertinent literature sources. This comprehensive exploration provides a snapshot of the envisaged enhancements and advancements within SCs, catalyzed by the integration of 6G capabilities. Section 8 presents some examples of SCs already implemented throughout the world. Finally, in Section 9, the paper distills the collective insights garnered from the preceding discourse. It encapsulates a list of currently existing challenges, implications inferred, and offers a set of comprehensive conclusions that underscore the pivotal takeaways from this comprehensive study. The abbreviations section at the end of the manuscript, lists all the acronyms in order of appearance with their descriptions, to facilitate reading.

## 2. Overview of Smart Cities

This section briefly describes the main characteristics of an SC, focusing on the enabling technologies (Section 2.1) and the management of involved resources (Section 2.2). Moreover, challenges and opportunities are shortly analyzed and discussed (Section 2.3).

### 2.1. Enabling Technologies

The ever-increasing computing power, together with the affirmation of 5G and the development of 6G, are key ingredients for the innovation of SCs, designed around people and sustainability. Blockchain, quantum computing, extended reality, and digital twins represent a non-exhaustive list of players that can inspire better cities. The key factors of SCs trace several closely interrelated areas.

- Artificial Intelligence (AI): AI has the potential to play a major role in the development of SCs by improving efficiency, sustainability, and the quality of life for citizens. AI-powered solutions can help SCs to reduce energy consumption, optimize traffic flow, and improve public safety. For example, AI algorithms can analyze traffic patterns to reduce congestion and air pollution, or to monitor energy usage in buildings to identify inefficiencies and reduce waste. SC initiatives can also benefit from AI-powered chatbots and virtual assistants, which can provide citizens with real-time information and support, such as directions or information on local services. Additionally, AI can be used to analyze data from various sources, such as social media, to understand citizens’ needs and preferences, and to make data-driven decisions that benefit the entire community.
- Data: Data analytics, data management, data storage, and data infrastructure are all critical components of the development of SCs. With the rise of the IoT and the increasing use of technology in cities, the amount of data generated is massive and growing every day. These data provide valuable insights into the functioning of a city, including traffic flow, energy consumption, and public safety. Data analytics allow cities to make sense of these data and make data-driven decisions to improve the efficiency and sustainability of the city. Proper data management and storage are critical components of an SC system, as they ensure that the data generated are secure and easily accessible for analysis [9,10]. A strong data infrastructure is also necessary for SCs, as it provides the foundation for all data-related activities. This includes the network, hardware, and software required to store, process, and analyze large amounts of data [11,12]. With the right data infrastructure in place, cities can take full advantage of the benefits of big data and data analytics, leading to a smarter and more sustainable future.
- Connectivity: Connectivity, 5G, 6G, latency, Wi-Fi 6, and IoT play a crucial role in the development of SCs providing the foundation for a smarter and more connected future. High-speed, reliable connectivity is necessary for the seamless functioning of SC systems, such as traffic management, energy management, and public safety systems. The roll-out of 5G and the upcoming 6G networks will provide faster and more reliable connectivity, allowing for the efficient transmission of large amounts of data generated by IoT devices. Low latency is also important in SCs, as it enables real-time decision-making and responsive actions. Wi-Fi 6 provides faster and more reliable Wi-Fi connectivity, allowing for the seamless functioning of IoT devices. IoT plays a key role in SCs by providing the data necessary for data-driven decision-making and by enabling the implementation of SC systems.
- Digital services: Simulated environments, digital twins, metaverse, virtual reality, robotics, and blockchain are all technologies that have the potential to revolutionize the development of SCs. Simulated environments provide a platform for cities to test and analyze new technologies and systems before they are implemented in the real world. Digital twins provide a virtual representation of a physical system, allowing for real-time monitoring and analysis. The metaverse is a virtual world that exists alongside the physical world, offering new opportunities for collaboration and innovation. Virtual reality and robotics offer new possibilities for training, simulation, and automation in SCs. Blockchain provides a secure and decentralized platform for the management and exchange of data, providing a foundation for the development of new and innovative SC systems. The development and implementation of these technologies in SCs is an exciting and rapidly evolving field, offering endless opportunities for innovation and progress.
- Cloud platforms: City platforms, cloud computing, cloud storage, edge computing, hybrid data storage, IaaS (infrastructure as a service), and PaaS (platform as a service) play critical roles in the development of SCs. City platforms provide a unified platform for the management of SC systems, such as traffic management, energy management, and public safety systems. Cloud computing and cloud storage provide scalable and flexible solutions for the storage and processing of large amounts of data generated by SC systems [13,14]. Edge computing enables the processing of data at the edge of the network, reducing latency and improving response times. Hybrid data storage combines the benefits of both cloud and edge computing, providing a flexible and scalable solution for data storage and processing. IaaS and PaaS provide the infrastructure and platform for the development and deployment of SC systems, respectively. These technologies provide a foundation for the development of new and innovative SCs, leading to a smarter and more efficient future.
- Internet of Things: IoT, machine-to-machine (M2M) communication, networked devices, and sensing are the actors in the development of SCs. IoT provides the backbone for the interconnectedness of SCs, such as traffic management, energy management, and public safety systems. M2M communication enables the seamless exchange of data between different devices and systems, providing a foundation for the efficient functioning of SCs. Networked devices, such as sensors and actuators, provide the data necessary for data-driven decision-making in SCs. Sensing, in particular, plays a crucial role in the collection and analysis of data, providing real-time information on a wide range of metrics, such as traffic flow, energy usage, and environmental conditions.

### 2.2. Resources Management

Achieving a certain number of elements that identify an SC necessarily involves the use and processing of a large amount of data. The coexistence of heterogeneous technologies, characterized by the presence of different radio access technologies (RATs), has consequently increased the demand for multimedia content and continuous amendments of network conditions. In this context, being able to select the best network solution has become crucial for guaranteeing a high quality of service (QoS). The operator’s limited network resources, where several access technologies coexist (e.g., 4G long-term evolution LTE, LTE advanced, and IEEE 802.11), need to be managed and shared in the best possible way. Load balancing is a prominent solution, dynamically scheduling the spatial varied traffic between cells, with the objective of avoiding traffic blocking in highly congested areas, leveraging the utilization of unused resources in decongested areas. Recently, several works in the literature have proposed the exploitation of load balancing for heterogeneous networks. The load balancing for multimedia streaming over the heterogeneous network algorithm, named adaptive real-time multi-user access network selection (ARMANS), is proposed in ref. [15]. Information such as traffic topology, service requests, and real-time traffic types dynamically identify the best candidate network. Thanks to a new priority-based approach, ARMANS is also able to reallocate resources in order to offset satisfaction, taking into account the priority, level of requested services, and characteristics of devices and applications. The need to consider energy consumption led the authors to develop an energy-aware device-oriented video delivery algorithm (E-ARMANS) [16], which leverages both traffic load and energy conservation, striving primarily to maintain high-quality standards for video content delivery. Adaptive real-time approaches have received a great deal of attention since it is critical to provide optimized solutions for media users within a SC. It is important to develop algorithms that take into account several factors for decision criteria, such as the network and user profile, the applications required, and the profile of the device used. These parameters are the input to a multiplicative exponential weighting (MEW) [17] approach that manages the access network selection.

To provide a high-quality experience for users using heterogeneous services, a solution to the complex problem of selecting the appropriate network medium for each type of user service is needed. In ref. [18], the authors proposed a network traffic type-based differentiated reputation (TYDER), a system that is able to discriminate the data delivery process according to the type, taking into account reputation in the context of traffic type requirements to improve delivery performance. Four traffic categories (i.e., gaming, browsing, video, and IoT) were considered for comparative tests, demonstrating how TYDER outperforms the classic solution in terms of all main performance metrics.

In the multimedia-based IoT (M-IoT), smart heterogeneous multimedia objects are allowed to collaborate together and with other internet-connected objects to simplify multimedia services and applications that are globally available to users. Nowadays, solutions performing access network selection for heterogeneous devices do not consider reputations related to a network in a specific instant. In ref. [19], the M-IoT ReMIoT architecture based on network reputation is introduced to improve the quality of multimedia content distribution. The solution comprises three distinct operational phases. The first phase is multimedia service detection, the second phase is network reputation detection, and finally, the M-IoT network selection. With the advent of 5G, it is possible to develop machine learning (ML) techniques for determining subgroups in order to target certain types of resources and content toward a narrow category of users. Currently, the burden of determining subgroups falls solely on the gNB transmitter node. In ref. [20], the authors developed a technique to relieve the computational burden on the gNB in evaluating the location and mobility of users, with the ultimate goal of determining the optimal modulation and coding scheme (MCS). ML techniques can help gNB establish the network configuration, accelerating the MCS determination and resource allocation to subgroups within 5G technology.

In accordance with sub-grouping techniques, the evolution of user devices, along with the growing use of social media, require the coexistence of on-demand and go-live multimedia contents, and the development of a combined broadcast/unicast distribution model, with efficient wireless access management as a key issue. The dual objectives include optimizing the load balancing between coexisting networks and providing adequate QoS to users. In ref. [21], ALOWGATH (i.e., ALOW + GATH) is presented, a solution that provides the joint use of (i) an additive logarithmic weighting (ALOW) algorithm that is able to combine information, such as the network load, packet delay, received signal strength, user equipment, and user credit budget; (ii) a cooperative game theory (GATH) algorithm to optimize ALOW.

Among the various application scenarios within SCs, smart grids are receiving increasing attention to both the flow of electricity and the flow of information related to the management and control of the electricity supply system. The domain of the International Communication Union (ICT) should not be perceived merely as a simple extension or modernization of electricity system equipment, but represent a fundamental requirement to support the monitoring, operation, and control of the distribution network. Similar to the systems seen above, smart grid monitoring and control require the management of extensive real-time data flow between the controlled equipment and the distribution management system. A dense network of sensors of advanced sensors and measurement systems as well as a communication network infrastructure are essential. In ref. [22], two different strategies for power grid monitoring and control are compared: an LTE-based centralized management approach and a 5G-based distributed management approach. The two types of communication systems are compared by considering their performance during fault management in a smart grid scenario.

### 2.3. Challenges and Opportunities

The digital transformation sweeping across society and the economy is a continuously evolving process that has been gathering momentum in recent years. It is driven by the rapid advancement of various technologies, especially AI, and the increasing availability of digital information. The process involves the use of digital technologies to transform existing economic and social structures and to create new opportunities for growth and innovation. An important element of digital transformation is the use of AI to automate and improve processes and services. AI is used to improve the efficiency of public services and optimize the delivery of services. For example, AI-based chatbots are being used to provide customer service to citizens and simplify the process of accessing services. AI can also be used to analyze data to detect fraud and identify potential areas of risk. AI-based algorithms can be used to provide personalized services and products tailored to user needs. The use of AI is also gaining traction in the area of SC solutions. AI can be used to optimize traffic patterns, reduce emissions, and improve public safety. AI can also be used to analyze data from the city’s various sensors to create insights and improve decision-making. In addition, AI-based systems can be used to monitor the environment and identify potential pollution sources. Despite all the benefits of SC-related projects, many challenges still pose serious problems when it comes to deployment, due to typical city requirements and differing interpretations of deployment concepts.

1. Public transport: The advantage gained by cities with extensive transportation systems is the result of numerous applications that simplify the experience of users using public transportation systems. Mobile applications enable real-time information management, providing users with solutions and viable alternatives in all those cases where delays may accumulate due to breakdowns, accidents, unforeseen events, or particular events within SCs. A very important role is attributed to IoT sensors installed on existing physical infrastructure, with minimal investment but huge benefits in the management of smart routine maintenance, scheduled in a timely manner and based on the continuous feedback received from IoT sensors. Intelligent transportation systems (ITSs) and the use of digital signage represent inputs for applications that simplify the user experience by providing real-time information about any delays or unforeseen events, enabling travelers to dynamically change their routes. Intensifying the use of IoT sensors by leveraging existing physical infrastructure provides a dual benefit, both for the end user and for the maintenance of monitoring and control facilities.
2. Traffic mitigation: Traffic mitigation and SCs are two closely related topics that are becoming increasingly important in urban planning. SCs are characterized by the use of technology to improve efficiency and reduce environmental impact, while traffic mitigation is the process of reducing traffic congestion and improving safety. Both are important elements of modern urban planning and can be used to create more livable cities. Applications that reduce road congestion are effective tools, especially in cities where public transportation and people driving their own vehicles are the main means of transportation. Car navigators provide a real-time overview of traffic conditions, alert drivers about the estimated time of arrival (ETA), and allow route rescheduling between the origin and destination. One of the most recent challenges involves smart parking apps that minimize search times and harmful emissions by directing drivers directly to available parking spaces.
3. Air quality: SCs use air quality sensors to detect dangerous levels of air pollutants, such as carbon dioxide, sulfur dioxide, ozone, and particulate matter. The sensors can detect small fluctuations in air quality and alert city officials to take action. SCs also use air quality modeling tools to predict potential air quality issues and develop plans to address them. Due to the growing problem of air pollution, several systems can now be used to monitor the air quality index (AQI) in urban areas. For example, in ref. [23], the authors developed smart bus stops, with smart bus shelters operated by a smart bus stop–social virtual object (SBS-SVO). The SVO features sensors to monitor air quality (temperature, humidity, barometer, and PM10) and a sniffer to assess crowding inside the bus. There is also an actuator that controls the screen that displays information to waiting passengers.
4. Solid waste reduction: Solid waste reduction, tracking, payments, and IoT technologies are important elements of modern waste management systems. These technologies can help reduce the amount of waste generated and track it in real time to ensure that it is disposed of properly. Payments are also a key factor in solid waste management, as they help waste reduction and tracking. Finally, IoT technologies can be used to monitor and manage the entire waste management system in real time. Waste tracking is also important for waste management, as it allows us to monitor how waste is disposed of and ensure that it is conducted properly. This can be achieved through GPS tracking or RFID tags, which allow us to monitor waste in real time. This helps reduce the risk of improper disposal and allows us to identify areas where more waste is generated.

SCs are characterized by smart heterogeneous devices cooperating with each other by exchanging regularly low amounts of data in the context of IoT. Lately, the scientific community has focused on the use of the IoT paradigm to allow the exchange of multimedia content. In ref. [24], a new algorithm is presented, enabling the best QoS and load balance in a 5G network context; named, mobility services user virtualization (MISSION) has been introduced, employing cloud computing and broadcasting to better manage the network load, the number of interactions, and energy consumption of user devices.

Another defining aspect of SCs involves true public green spaces, biodiversity, mountain preservation, and coastal erosion monitoring and control. In ref. [25], the authors presented a coastal crowding monitoring system based on the social IoT (SIoT); this is a promising solution that safeguards coasts from the erosion process (mainly due to natural factors, urbanization, and massive tourism) and manages (in a “smart and green” fashion) a large number of tourists. SIoT is a new paradigm that defines a social network between objects (i.e., devices) that are capable of autonomously establishing relationships with other things according to specific rules. In this context, all involved devices in the monitoring system (i.e., cameras, sensors, traffic lights, or smartphones) can collect and exchange information. The proposed system, carried out and installed in Cagliari (Italy), evaluates the occupational state of a beach, considering the crowding level, environmental data (collected by devices installed at specific points of the city), and real-time feedback sent by users, thanks to the use of a specific application.

Similar work involved pedestrian and vehicular mobility monitoring and control. Ever-increasing urbanization represents one of the key aspects in the field of mobility in the study of SCs. The phenomenon related to the flow of people moving within the city is gaining attention. Indeed, the phenomena of road congestion and overcrowded areas are on the rise. In ref. [26], the authors employ tools for the monitoring phase through Wi-Fi-sniffing systems and video cameras to classify vehicles, people, bicycles, and so on. Real-time monitoring allows citizens to know road conditions in advance with a high level of reliability, overcoming the limitations of a single technological system that provides inaccurate monitoring. The goal is to perform a real-time monitoring procedure to improve the lives of citizens by providing advanced knowledge of traffic conditions. The joint use of two technologies overcomes the limitations of using only one technology. Initially, each technology is performed individually, and the results obtained are combined using NNs with a high degree of accuracy.

The SC paradigm plays a primary role in the implementation of sustainable solutions in the field of urban mobility, both public and private. The SIoT paradigm adds a relational connotation between objects typical of human relationships. Objects operate as equals and request/provide information to each other with a view of providing IoT services to users while maintaining their individuality. The social relationship between objects enables the design of solutions to improve the exchange of information between network nodes in terms of security from malicious attacks external to the so-called social network of objects. In this context, a new SIoT solution for the SC is presented in ref. [27]; private and public vehicles, together with pedestrians, are involved in real-time data collection to improve the city’s road system in order to suggest new directions and information to citizens to better organize how they experience the city. The developed architecture is equipped with AI, which processes the collected traffic data and, using ML techniques, evaluates the directions and flows taken by vehicles and pedestrians on a daily basis. The combined effect of the large-scale adoption of mobile communications, the recent advent of low-power wide area networks (LPWANs) promoted by the IoT, and the unprecedented diversification of next-generation networked applications offer new opportunities for further advances in wireless communications. The growing demand for reduced energy consumption has enabled the development of innovative solutions and low-power network communication technologies to be jointly deployed in a real-world scenario in an SC environment [28,29,30].

The use of energy-efficient technologies and communications, along with emerging areas, such as augmented reality (AR) and virtual reality (VR), monitoring attention [31], and improving safety [32], are just some of the challenges facing emerging technologies in delivering new services to citizens. In ref. [33], the authors propose a multi-hop communication solution (e2McH) that performs energy-efficient multi-hop communications over LPWAN, via narrowband technologies. The push for more energy-efficient networks, where receiver systems cannot be installed, has necessitated the development and testing of single- and multi-hop configurations, demonstrating potential energy savings of up to 15%. Multimedia applications in SCs provide users with innovative sensory experiences, for example, for the fruition of cultural heritage based on imaging [34]. They have recently been joined by multisensory experiences; that is, providing users with realistic multimedia content. In this way, the user’s sense of immersive reality can be enhanced by adding multiple sensory effects to conventional media through the stimulation of five senses (i.e., taste, sight, touch, smell, and hearing). In refs. [35,36], for example, a study was conducted on smart homes; it can be replicated in other SC contexts. In addition to high-quality audio-video content, additional effects were provided by exploiting traditional devices (e.g., air conditioning, lights, etc.), equipped with appropriate smart features, as opposed to ad hoc devices often used in other applications, such as gaming systems [37]. The IoT paradigm has been widely adopted to connect smart devices through an IoT-based architecture for delivering multisensory media to users within a home entertainment scenario. Obviously, the synchronization between media and devices defined within the system architecture plays a very important role in terms of the quality of experience (QoE).

## 3. Evolution of Mobile Networks before 6G

Notably, 3G and 4G wireless technologies were mainly driven by the demand for data services over the internet. The development of 5G was driven by new emerging traffic types and data services, characterized by different requirements and issues to be solved, and an anticipated increase in capacity of up to 1000 times in the next decade on the network side [38]. Radio access, network slicing, and edge computing are three important concepts in the field of telecommunications and networking [39]. Radio access refers to the technology used by wireless devices to connect to a cellular network. This can include technologies such as 3G, 4G, and 5G, and determines the speed and reliability of wireless data transmission. Network slicing is the process of dividing a single physical network into multiple virtual networks, each with its own unique characteristics and requirements. This allows different applications and services to operate independently on the same physical infrastructure, which improves network efficiency and flexibility. Edge computing refers to the practice of processing and analyzing data closer to the source of that data, rather than sending the data to a centralized data center or cloud. This reduces latency and improves the speed of data processing, which is particularly important for applications that require real-time processing, such as autonomous vehicles or industrial automation. Together, these three concepts help drive innovation in the telecommunications industry, enabling the development of new applications and services that require high-speed, low-latency, and highly reliable network connections.

Network operators aim to satisfy the growing traffic demand by deploying cells of various sizes as elements of heterogeneous infrastructures, which need additional intelligence to be operative. The 5G networks are expected to support multiple radio access technologies with overlapping coverage deployed as part of a single multi-radio heterogeneous network, supporting end-to-end network architectures and protocols that seamlessly combine multiple radio access technologies together into a single virtual radio access network (RAN), in a transparent manner to end users [40]. Emerging technologies, such as software-defined networking (SDN) and network function virtualization (NFV) have been proposed for applying intelligence toward 5G communications [41]. SDN has arisen as a reaction to the limitations and intricacies inherent in conventional network architectures. The core concept of SDN involves consolidating authority over network devices within a logically centralized (software) controller, distinct from the data plane. This division between the control and data planes is achieved through an accessible programming interface connecting the data plane switches to the SDN controller. This dissociation empowers the control plane to evolve autonomously from the data plane, facilitating swifter innovation, as software frequently outpaces hardware in terms of innovative velocity. Additionally, the notion of logical centralization holds the potential to streamline network operation and administration, by offering a singular focal point for evaluating the ramifications of managerial actions, with the ability to potentially reject actions that might breach operational constraints. Presently, OpenFlow, the established SDN protocol, hinges on a straightforward match–action paradigm, endowing considerable flexibility, such as in traffic engineering, flow definition, and even in-band network control functionalities [42]. The incorporation of SDN into existing networks gives rise to operational and implementation challenges. Within this context, ref. [43] presents an innovative strategy—denoted as a smart approach—to enhance the performance of dependable and time-critical flows within hybrid SDN-based fog computing (FC) IoT systems (IHSF). The outcomes of the conducted tests demonstrate that the proposed IHSF solution outperforms the pre-existing approach in terms of network observability time, flow disruptions, end-to-end latency, and packet delivery ratio. To enhance communication infrastructures in SCs, the integration of two emerging technologies, FC and SDN, is gaining traction. This combined SDN-based FC architecture aims to meet IoT application needs, emphasizing easy management, scalability, reliability, and low latency. While earlier SDN-based FC traffic approaches considered quality of service (QoS) constraints when computing routes between IoT devices and fog servers, they overlooked link reliability [44].

Initially, several organizations (e.g., the European Telecommunications Standards Institute (ETSI) and the third-generation partnership project (3GPP)) started programs to individuate key technologies for 5G [45,46,47]. A collective thought was to consider higher spectral efficiency, lower latency, traffic asymmetry, hotspot traffic, and energy efficiency as main requirements [48]. The International Telecommunication Union (ITU) International Mobile Telecommunications (IMT) 2020 focus group, established in May 2015 [49], suggesting potential technological improvements, focusing on support for data rates of up to 20 gigabits per second, the ability to allow massive armies of devices to connect in a small area, and reduced energy consumption [50]. Considering the spectrum use, frequencies below 6 GHz were considered suitable for macro-coverage (i.e., a radius of up to 2 km). For frequencies of up to 30 GHz, about 2.5 GHz could be made available for micro-coverage. Frequencies from 30 to 90 GHz (i.e., visible light) were considered suitable for front-hauling, back-hauling, as well as local deployments (i.e., within a 10 m radius). In this range, about 40 GHz could be allocated for massive machine communications [51].

Flexible adaptation should be a main requirement to allow communication between vehicular devices moving fast and operating at high frequencies. Filter bank-based multi-carrier (FBMC) and universal-filtered multi-carrier (UFMC) were considered as enabling technologies to allow the coexistence of new services performing efficient utilization of narrow frequency bands. Moreover, non-orthogonal multiple access (NOMA) and sparse code multiple access (SCMA) were two other promising methods used to improve spectrum utilization, especially for dense networks and multicast services. Multiple-input multiple-output (MIMO) and beam-forming techniques were strengthened to increase coverage capacity and limit interference, exploiting the coexistence of transmitting and receiving antennas [51]. Another potential solution is the utilization of cell-free massive MIMO (CF mMIMO), which involves multiple wireless access points working together to process coherent signals and serve users collectively. However, achieving both massive connectivity and high SE simultaneously is problematic due to limited pilot resources. To address this issue, ref. [52] proposes a new framework for energy self-sustainability (ESS) Internet of Everything(IoE) networks that decouple user activity detection (UAD) and channel estimation. A further solution is proposed by ref. [53], i.e., a novel framework for ESS IoE networks that decouples UAD from channel estimation. In this approach, a UAD detector based on deep convolutional neural networks (CNNs) is introduced, along with an initial access scheme and a scalable power control policy, enabling the practical and scalable implementation of CF mMIMO.

The 5G network was considered to be heterogeneous, consisting of macrocells along with a large number of low-power nodes. In this context, an industry proposal was made to improve spectral efficiency, combining the LTE standard (i.e., 4G) with Wi-Fi technology, leading to the approval of 3GPP in new projects, to develop new releases (i.e., from release 8 to 14) and interworking between LTE-A and Wi-Fi. This new LTE in an unlicensed band (LTE-U) has the physical layer topology to access the Wi-Fi spectrum, specifically the 5 GHz band. The main side-effect involved the coexistence in a very congested band. In ref. [54], the authors proposed two coexistence models, implementing modifications at the RAN and backbone sites to enable advanced features typical of 5G networks. Device-to-device (D2D) communication, which allows devices to share their radio access connections, emerged as a potential solution to reduce the cost of local service provision and address the rising density of networks. This change was in line with evolving trends in the telecommunications market [55]. Indeed, the centralized architecture of mobile networks required some adjustments to support the growing traffic. For this reason, traffic generated in the RAN was conveyed by serving gateways to packet data network gateways, performing as a gateway between the operator’s network and external IP networks. Considering the core, the idea was to route traffic without unnecessary core links, allowing direct communication between devices. Increasing flexibility and adaptation capacity were considered priorities, forecasting that 5G networks would cater to a growing number of heterogeneous devices with different requirements (i.e., IoT). Accordingly, 3GPP started developing solutions for the evolved packet system (EPS) in order to bypass the operator’s backhaul and core infrastructure, to allow for a local connection to internet services, preventing locally generated traffic from being rerouted to the core network [56].

Full-duplex systems were indicated as a promising solution in order to provide significantly higher data rates than conventional half-duplex communication systems, reducing the latency by simultaneously receiving feedback signals from the receiver during transmission [48,57]. In parallel, due to increasing global carbon emissions and alarming pollution levels, especially in dense cities all over the world, energy saving was recognized as a primary issue worldwide. The GreenTouch consortium [58], founded in 2010 as an open global research consortium, presented its final results in its “Green Meter” research study [59]. Additionally, the Groupe Speciale Mobile Association (GSMA) set a target to reduce CO_2_ emissions per connection by more than 40% by 2020. These fundamental facts have led to the notion of bits-per-joule energy efficiency, which is defined as the amount of information that can be reliably transmitted per joule of consumed energy; it became a key performance indicator for 5G networks [57,60]. In ref. [61], energy efficiency was treated as a performance parameter and design constraint for communication networks; the authors pointed out interesting research directions and highlighted that many technical, regulatory, policy, and business challenges remained to be addressed before the ambitious 1000-fold energy-efficiency improvement goal could be reached.

Considering the percentage of total information and communications technology (ICT) energy consumption (i.e., 25%) [62], 5G was also expected to introduce green communication technologies, taking into account the growing necessity for infrastructure and devices [63]. Several technologies were proposed to improve capacity, coverage, and energy efficiency, such as heterogeneous networks, software-defined cellular networks, and M2M communications [64]. In ref. [65], the authors proposed the utilization of a cognitive network to lease additional spectra outside the licensed cellular bands, being the cognitive radio resource characterized by its potentially broad bandwidth, low transmit power, and low reliability. They showed an energy efficiency-spectrum efficiency trade-off study to provide direct guidelines to the OPEX (operator experience) management of cognitive cellular networks, and proposed different architectures and usage scenarios of cognitive cellular networks. Table 1 summarizes the main features and benefits introduced by 5G.

## 4. The Role of 5G in Smart Cities

We should note that 5G technologies in SCs play very important roles in several aspects: basic internet, broadband technology, video streaming, high-speed internet, connection to the world, and vertical industries. In ref. [66], the authors discuss the impacts and implications of 5G on ITSs from various dimensions. They present an overview of the technological context and economic benefits of 5G and how the main vertical sectors will be affected in an SC, namely energy, healthcare, manufacturing, entertainment, automotive, and public transportation.

Radio access technologies are the backbone of modern wireless communication systems, enabling high-speed data transmission and reliable connectivity for mobile devices. With the advent of 5G technology and the next generation of cellular networks, radio access technologies are evolving to provide even faster speeds, lower latency, and increased network capacity. One of the key features of 5G is its use of higher-frequency bands, including millimeter wave (mmWave) spectrum, which can provide significantly faster data speeds than previous generations of cellular technology. However, these higher frequencies have shorter ranges and are more susceptible to interference, which requires the use of advanced technologies, like beamforming and massive MIMO to ensure reliable connectivity. Ultra-dense networks (UDNs) are key technological enablers of 5G; however, the densification of small cells from multiple RATs poses challenges to network performance [67]. Therefore, an efficient RAT selection mechanism is necessary to choose the best available technology. In ref. [68], the authors propose a new context-aware radio access technology (CRAT) selection mechanism that considers both user and network contexts to make an appropriate RAT selection. A mathematical model based on the analytical hierarchical process (AHP) and technique for order of preference by similarity to the ideal solution (TOPSIS) was developed, and the proposed CRAT mechanism was implemented and validated in a simulation environment. The CRAT mechanism outperforms the conventional approach in terms of the number of handovers, average network delay, throughput, and packet delivery ratio in SC environments, such as shopping malls and urban areas. The 5G waveform features more flexibility, multiple access support, the ability to co-exist with different waveforms, low latency, and compatibility with massive MIMO and mmWave communications. While orthogonal frequency-division multiplexing (OFDM) has been the dominant technology in many existing standards and is still a favorite for broadband communications in 5G RAN, in ref. [69], the authors explore the merits and shortcomings of OFDM for 5G RAN scenarios, including enhancing waveform characteristics, such as out-of-band leakage and peak-to-average power ratio, as well as methods to reduce the time and frequency redundancies of OFDM, such as cyclic prefixes and pilot signals.

The development of smart communities relies heavily on communication through the IoT. For the IoT to continue to grow rapidly, dependable wireless networks are essential. One solution to this challenge is the use of RAN [15] or cloud RAN (CRAN) [70] in the evolving 5G cellular system, which incorporates cloud computing technology in the radio access network. CRAN provides greater scalability, flexibility, and performance, enabling 5G to connect a vast number of IoT devices that are crucial for SCs. The focus of this research is on reducing latencies in IoT communication by addressing the load balance (LB) problem in CRAN. The study investigates eight practical LB algorithms in a CRAN environment, using actual cellular network traffic characteristics provided by Nokia Research. Results indicate that the simple, lightweight queue-based LB is almost as effective as the much more complex waiting-time-based LB. This research has significant implications for 5G networks and their ability to serve as the backbone of IoT communication in smart communities, as well as for other distributed systems.

Another important aspect of 5G RATs is the concept of network slicing, which allows service providers to create customized virtual networks for different types of applications and services [71]. For example, a network slice could be created specifically for autonomous vehicles, with low-latency connections and high reliability, while another slice could be optimized for streaming video, with high data speeds and large bandwidth allocations. Overall, 5G RATs are expected to enable a wide range of new applications and services, from AR and VR to the IoT and SCs. As the technology continues to evolve and expand, we can expect to see even more innovative uses of wireless communication in the years ahead. The idea behind network slicing is to divide a single physical network into multiple virtual networks, each with its own unique set of characteristics and capabilities. This allows for the more efficient use of network resources, as well as better control and management of network traffic. In the context of IoT, network slicing can be used to automatically allocate network resources based on the specific needs of each device or application. This is particularly important in SCs, where IoT devices are expected to play a significant role in areas such as traffic management, public safety, and environmental monitoring. An automatic network slicing system would be able to dynamically allocate network resources to IoT devices based on their individual requirements, without requiring manual intervention from network administrators [72]. This would enable more efficient use of network resources, as well as better support for a wider range of IoT devices and applications. In ref. [73], the authors suggest the optimization of SCs components for 5G network slicing, also involving the development of a network architecture that can dynamically allocate network resources based on the specific needs of different SC applications and services. This means taking into account the different requirements of each application or service, such as latency, bandwidth, and reliability, and ensuring that the network can allocate resources accordingly. One example of this is the deployment of smart traffic management systems that are optimized for 5G network slicing. These systems would be able to dynamically allocate network resources based on traffic conditions, ensuring that critical information is delivered quickly and efficiently.

Edge computing involves processing data and performing other computational tasks closer to the source of the data, rather than sending them all to a central data center or cloud. This can be achieved using edge devices, which are located closer to the edge of the network and can process data in real time. When combined, edge computing and 5G can provide a powerful platform for creating new applications and services that were previously impossible. For example, real-time video analytics can be performed at the edge of the network using edge devices, with the processed data transmitted over 5G networks. This can enable a wide range of applications, such as remote health monitoring and surveillance. Another example is the use of 5G and edge computing for autonomous vehicles. By processing sensor data and other information at the edge of the network, autonomous vehicles can make real-time decisions, increasing safety and efficiency.

The presence of a cloud in the IoT system of an SC can result in high energy consumption and network delays. To address this, edge computing, which is based on a cloud computing framework, has emerged as a solution that moves computation, storage, and network resources closer to the data source. However, the efficient use of energy while maintaining delay limitations is a critical issue in edge computing when executing tasks generated by IoT systems. In ref. [74], a multi-criteria optimization approach is studied for resource allocation with distributed edge computing in IoT-based SCs. A three-layer network architecture for IoT-based SCs is proposed, and an auctionable approach-based edge resource allocation scheme is presented to ensure efficient resource computation for delay-sensitive tasks. Edge computing offers several benefits [75] in SCs and the IoT, including:

- Reduced latency: Edge computing moves computation and data storage closer to the edge of the network, which reduces latency and provides real-time processing of data. This is particularly important for applications that require real-time processing, such as traffic management, video surveillance, and emergency response systems.
- Improved security: Edge computing can enhance security by enabling data to be processed and stored locally, rather than being transmitted to a central data center or cloud. This reduces the risk of data breaches and other security threats.
- Increased scalability: Edge computing allows for greater scalability by distributing computing and storage resources across multiple edge devices. This enables more efficient use of resources and improves the overall performance of IoT systems and SC services.
- Lower costs: Edge computing can reduce costs by minimizing data transmission and storage requirements. This can be particularly beneficial for organizations operating in remote or underdeveloped areas where internet connectivity may be limited or expensive.
- Enhanced reliability: Edge computing can improve the reliability of IoT systems and SC services by enabling them to function even in the event of network failures or disruptions. This can be critical for applications such as public safety and emergency response.

The virtualization technology proposed in ref. [76] requires security and technology extensions to support a unified view of sliceable and heterogeneous devices and radio technologies such as LTE, 5G, and Wi-Fi. The security of the system is improved by deploying robust compute node authentication, monitoring, and geo-tagging, while wireless connectivity is extended through an innovative multiple RAN controller approach for the management and control of heterogeneous radio resources.

## 5. 6G—The Emerging New Standard

The 5G of cellular networks has revolutionized the way people communicate and connect together. With its ultra-fast speeds, low latency, and massive connectivity capabilities, 5G has opened up new opportunities for businesses, industries, and consumers alike. However, as technology continues to evolve, researchers and industry experts are already looking ahead to the next generation of cellular networks, which is 6G.

Several reasons can justify the development of a new wireless technology generation. Firstly, when the run to 5G finishes, all the industries interested in telecommunication business will need a new innovative product to push; they will focus on new methods to offer new services or improve the old ones. At the IEEE Future Directions Committee Meeting [77], within the Industry Advisory Board (IAB), the evolution of wireless systems was discussed, with a focus on 5G; a draft of the 6th generation as a significant evolution of 5G was traced due to its ability to self-aggregate networks of different types, satisfying the requests of resources that will present themselves in a dynamic way.

The mobile network trend (5G) has shifted in its primary goal compared to previous networks; the main goal has shifted from enabling users to connect wirelessly to the internet to enabling huge numbers of users and devices to connect seamlessly to SCs (IoT) by 2020 and beyond [78]. At the World Radiocommunication Conference (WRC) 2015, the main goal was to add an additional spectrum for mobile communications below 6 GHz. However, the massive mobile traffic growth of global mobile traffic cannot be met by adding an additional spectrum band below 6 GHz, as promoted at the 2015 WRC. Therefore, it was decided at the 2019 WRC that additional frequency bands above 6 GHz will be needed to achieve multi-Gbps data rates. Although 6G is still in the early stages of development, it is expected to offer even faster speeds, lower latency, and more advanced capabilities than 5G. Some of the key areas of focus for 6G research include:

- THz frequencies: 6G is expected to utilize higher frequency bands than 5G, including terahertz frequencies, which have the potential to offer even faster data rates and lower latency.
- AI: 6G is expected to integrate AI more deeply into its design, enabling more intelligent network management and a range of new applications and services.
- Quantum computing: 6G is expected to leverage the power of quantum computing to enable new capabilities, such as secure communications and advanced sensing.
- AR and VR: 6G is expected to enable more seamless and immersive AR and VR experiences, enabling new applications in areas such as gaming, education, and healthcare.

While 6G is still several years away from becoming a reality, the groundwork is already being laid for this next generation of cellular networks. As researchers and industry experts continue to push the boundaries of what is possible, we can expect 6G to offer even more exciting opportunities for innovation and growth in the years to come.

The 5G network aims to have a common platform for all radio access technologies (gsm, LTE, Wi-Fi, etc.). In Table 2, a comparison is made between 5G and 6G technologies, considering the main technological and application differences.

We should note that 6G proposes to integrate 5G with satellite networks (i.e., telecommunications and GPS navigation) in order to reach connection speeds up to 11 Gbps. Nano-antennas will be used for the development of the Internet of Things. Global coverage and high-speed internet connectivity are the main objectives of 6G technology. Moreover, 6G networks will embed planning into the network itself, meaning that the network will become aware of the way it is being used, what is actually required by its users at this specific moment, and what is likely to be required at a later time; it will be able to plan for its evolution by reconfiguring its resources and asking vested parties to provide additional resources, leading to convincing reasons and convincing business plans. Driving trends of emerging 6G are summarized in ref. [79]:

- The convergence of communications, computing, control, localization, and sensing (3CLS): Computing, control, localization, and sensing in addition to current wireless communication.
- Smart reflective surfaces and environments: Driven by smart reflective surfaces that serve as walls, roads, doors, and entire buildings, helping to maintain a line of sight and obtain a quality signal with minimal loss.
- Massive availability of small data: Shifting from centralized big data to massive distributed small data.
- More resource availability (i.e., bits, spectra, and reliability): Higher frequency spectrum (THz), reaching 1 Tb/s.
- Self-sustaining networks: AI to facilitate intelligent wireless networks that are self-sustaining.
- Ubiquitous connectivity that encompasses air, ground, and sea: Integrating the space–air–ground–sea mode to facilitate wireless communications in flying vehicles, XR, brain–computer interface (BCI), and more.

## 6. 6G for New Smart Cities

As the demand for advanced technological solutions in SCs continues to grow, there are many discussions around the potential for 6G networks to provide the infrastructure needed for these cities of the future. While 5G networks have made significant strides in improving connectivity and supporting the IoT, 6G networks are expected to provide even faster speeds, lower latency, and greater capacity, making them ideal for supporting the complex systems and devices in SCs.

With the help of 6G networks, new SCs could leverage a range of advanced technologies, including autonomous vehicles, smart grids, and remote healthcare. Additionally, 6G networks could support innovative applications, like augmented reality, virtual reality, and immersive gaming experiences, creating new opportunities for businesses and residents in these cities.

However, the development of 6G networks is still in its early stages, and it may take several years before they become widely available. In the meantime, researchers and industry experts continue to explore the potential benefits and use cases for 6G networks in new SC developments. Representative use cases in the context of SCs have been individuated and are presented as follows [80]:

- Intelligent transport and logistics: It is expected that in 2030 and beyond, autonomous vehicles and drones will facilitate the safe, efficient, and eco-friendly movement of people.
- Connected robotics and autonomous systems: Self-driving vehicles, which perceive their surroundings by combining a variety of sensors (e.g., light detection and ranging (LiDAR), radar, GPS, and sonar).
- Global ubiquitous connectivity: Maritime and river applications require network coverage for both the water’s surface and the depths below to monitor and manage ecosystems, especially in cities with rivers or ports where resources might be difficult to access.
- Pervasive intelligence: The growing presence of connected equipment, such as robots, smart cars, or drones, will bring out new applications that will require new methods or the updating of computationally intensive AI technologies (e.g., computer vision, simultaneous localization and mapping (SLAM), face and speech recognition, natural language processing, and motion control).

**Figure 1:**
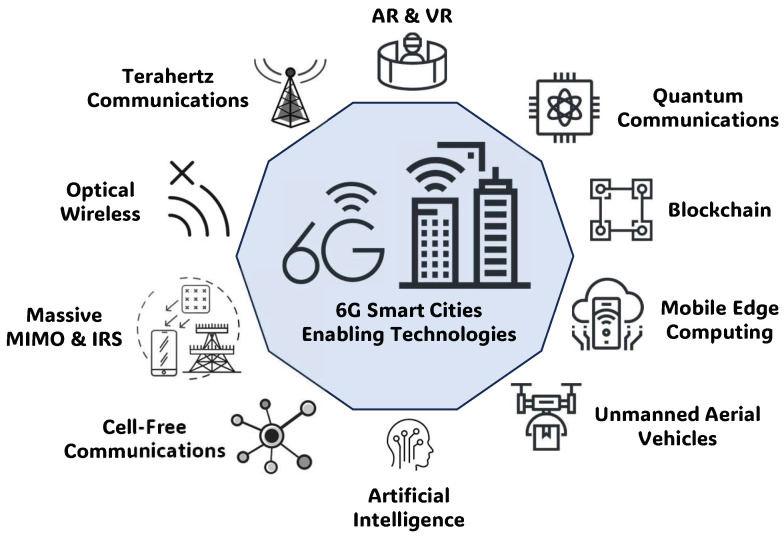
6G, smart cities, and enabling technologies.

Enabling technologies that are useful for developing these new applications are summarized in Table 3 and illustrated in Figure 1.

In more detail [81]:

- Terahertz communications: According to the recommendations [82], the 275 GHz–3 THz band range will be assigned to cellular communications and added to the mmWave band (30–300 GHz), potentially increasing more than 11 times the total band capacity. The critical issue of THz interfaces will probably lead to the use of highly-directional antennas.
- AI: AI can reduce the processing delay for communications and improve time-consuming algorithms (e.g., for handover or network selections) [83].
- ML: These techniques can provide solutions for resource management and mobility management challenges in 6G wireless networks, as with other technical challenges, such as resource allocation, task offloading, and handover management [84].
- Massive MIMO and intelligent reflecting surfaces (IRSs): An IRS [85] is a recent hardware technology (also known as a meta-surface) that provides energy-efficient green communication; it consists of many reflecting diode units, reflecting incident electromagnetic signals with an adjustable phase shift.
- Blockchain: It combines a distributed network structure with a consensus mechanism and advanced cryptography, thereby increasing security by eliminating single-point failures. The main weaknesses in 5G are throughput (i.e., 10∼1000 transactions per second) and the interoperability levels between different platforms. New consensus algorithms together with innovative architectures and sharing techniques will reduce these limitations in 6G networks.
- Quantum communication: The emerging paradigm of quantum computing [86] is considered one of the main enablers of 6G, improving its security by using a quantum key based on the quantum no-cloning theorem and uncertainty principle. Data are encoded in the quantum state using photons or quantum particles and cannot be accessed or cloned without tampering with them due to the quantum principles. Moreover, quantum communication improves throughput thanks to the superposition inherent in qubits.
- Cell-free communication: Conventional cellular and orthogonal communications will be shifted toward cell-free and non-orthogonal communications. Users will be able to move from one network to another network, choosing and selecting the best one automatically (i.e., between the available communication technologies), solving issues related to handover failures and delays, data losses, and the ping-pong effect prevalent in cellular networks.
- Unmanned aerial vehicles: UAVs carry onboard base stations (BSs) to offer cellular connectivity; they have specific features, such as easy deployment, strong line-of-sight links, and degrees of freedom with controlled mobility. UAVs are valuable during emergencies (e.g., natural disasters).
- Mobile edge computing (MEC): Due to the distributed massive cloud applications, mobility-enhanced edge computing (MEEC) has become a crucial part of 6G technology [87]; it addresses challenges in cloud-based scenarios, where the long-distance transmission of data from end devices and edge servers to the cloud incur great latency and security risks and consume a great amount of bandwidth.
- Optical wireless: This technology will be extensively used in several applications, such as vehicle-to-everything (V2X) communication and underwater optical wireless communications, providing very high data rates and low latency. LiDAR [88] is a promising technology for very high-resolution 3D mapping in 6G communications. Also, microLED technologies and spatial multiplexing techniques will be mature and cost-effective in 2026 [81].
- Free-space optical (FSO) communications: Allow for ultra-fast data links that can be applied in a variety of 6G applications, such as heterogeneous networks with enormous connectivity and wireless backhaul for cellular systems [89].
- AR and VR: 6G is expected to enable more seamless and immersive AR and VR experiences, enabling new applications in areas such as vehicular communications and SC security.
- Energy harvesting: The massive growth of devices and data traffic is leading to great energy demands for 6G; energy harvesting is a promising technology used to mitigate the dilemma between the high energy demands and limited battery capacities [90,91].
- Low-orbit satellite: With the rapid development of satellite communication technologies, the requirements for 6G can be satisfied by incorporating space-based and ground-based cellular networks [92]. Low-orbit satellite constellations play a crucial role for space-ground interconnections, ensuring full-coverage broadband services for ground users.

## 7. 6G-Enabled Smart Cities

One of the key concepts of 6G-enabled SCs regards the integration of advanced communication technology, specifically 6G networks, into the development and management of SCs. An SC utilizes various digital technologies and data-driven solutions to enhance the quality of life for its residents, improve infrastructure efficiency, and promote sustainable development. Moreover, 6G networks can facilitate the development of intelligent infrastructure, such as connected sensors and devices, enabling better monitoring and management of urban resources. This includes smart waste management, optimization of water and energy usage, and intelligent urban planning based on data analytics. Furthermore, 6G-enabled SCs are expected to promote inclusivity and improve the quality of life of citizens by enabling better access to services, such as e-healthcare, online education, and digital governance platforms.

In this section, 6G-enabled SC solutions proposed in the literature are briefly described and grouped into subsections, considering fundamental enabling technologies summarized in Table 3 and use cases. Table 4, at the end of the section, summarizes the correspondence between enabling technologies for SCs in 6G and the works in the literature that describe their use.

### 7.1. Terahertz Communications

In ref. [93], the authors assert that 6G needs AI-enabled optimization. Traditional approaches are characterized by prior knowledge and statistical analysis but are ineffective due to the elapsed time from the analysis to the decision-making [94]. AI-enabled 6G network protocols and mechanisms, with their employed self-learning ML and deep learning (DL), algorithms are proposed in order to solve several issues in networking.

The concept of futuristic SCs and the role of 6G network technology in their development are discussed in ref. [95], highlighting the need for dense and AI-centric cities, as well as the requirement for massive device connectivity and data traffic. A 6G network is seen as the solution for these futuristic cities, offering high bandwidth and low latency using terahertz waves. However, the short-range and atmospheric attenuation of terahertz waves present challenges to the 6G network. The study proposed a conceptual terrestrial network architecture called the nested Bee Hive, designed to address the needs of futuristic SCs. Simulations using different pathfinding algorithms have evaluated the performance of this architecture and established the dynamics of communication in a 6G environment.

**Table 4:** The 6G enabling technologies for SCs in the literature.

	THz	AI	M-MIMO	BC	QC	UAVs	MEC	OW	VR/AR
**[93]**	x	x	x	x	x		x		
**[95]**	x	x	x						
**[96]**	x		x						
**[97]**	x								
**[98]**	x								
**[99,100,101,102,103]**		x							
**[104]**		x							
**[105]**		x							
**[106]**		x	x						
**[107]**			x						
**[108]**				x		x			
**[109]**		x		x					
**[110]**		x		x					
**[111]**		x		x					
**[112]**		x		x					
**[113]**		x		x					
**[114]**					x				
**[115]**					x				
**[116]**		x			x				
**[117]**	x	x			x			x	
**[118]**						x			
**[119]**						x			
**[120]**		x				x			
**[121]**						x			
**[122]**						x			
**[123]**							x		
**[124]**							x		
**[125]**							x		
**[126]**							x		
**[127]**							x		
**[128]**							x		
**[129]**							x		
**[130]**							x		
**[131]**									x
**[132]**									x

Ref. [96] suggests overcoming the limitations of current THz antennas and IRS by only considering single-carrier modulation with a small array scale through the use of ultra-massive MIMO. The THz links are subject to high-loss connectivity. In ref. [97], the authors propose a cost-effective method to dynamically optimize the transmission path using reconfigurable intelligent surfaces (RISs), constructed by embedding active elements into passive metasurfaces, proposing metasurfaces with tunable functions, such as pixel-level amplitude modulation and wide-range phase coverage for beam steering. The utilization of ITSs and autonomous driving in the 6G era heavily relies on the exchange of massive amounts of information with ultra-wide bandwidth, high reliability, and low latency to ensure safety and provide a seamless experience. However, signals in high-frequency bands, such as millimeter wave (mmWave) and THz, can encounter obstacles that hinder their propagation. To tackle this issue, IRSs have garnered significant attention due to their ability to intelligently reflect signals and alter their propagation directions, thereby creating smart radio environments. IRSs offer advantages, such as easy deployment and cost-effectiveness, making them promising technologies for ground and aerial vehicular networks. They can amplify signal strength, bolster physical layer security, and enhance positioning accuracy [98].

### 7.2. Artificial Intelligence

Adaptive radio communications are technologies that have been around under various forms, such as the cognitive radio concept, for the past 20 years. The next step, the transformation to intelligent radio, can be supported in the 6G context by using advanced AI algorithms, in order to dynamically adapt radio communications to a specific radio environment.

As the data traffic grows in SCs, different modulation methods are being employed in communication systems for efficient and effective data transmission. Modulation recognition is crucial in signal demodulation and decoding, especially in applications such as interference identification, signal recognition, spectrum monitoring, and threat assessment. For instance, methods such as convolutional neural networks (CNNs) and recurrent neural networks (RNNs) for modulation recognition over additive white Gaussian noise and Rayleigh fading channels were presented in ref. [99]. A CNN-based federated learning approach was proposed in ref. [100], enabling differential privacy for modulation recognition in order to assure the privacy and security of transmitted data.

Apart from the previously presented modulation classification techniques, traffic classification into different classes is another technique to ensure the QoS, enable pricing control, improve resource management, and improve the security of SC applications. In ref. [101], the authors proposed a Tree-RNN to classify network traffic into 12 different classes, thanks to a tree structure that divides the large classification problem into smaller ones, with each class represented by a tree node. Ref. [102] describes a hybrid RNN and CNN-based network to classify traffic from IoT devices and services. CNN layers extract complex network traffic features automatically from the input data, eliminating the feature selection process used in classical ML.

Channel estimation is the process of estimating the characteristics of the communication channel to recover the transmitted information from the channel effect. In ref. [93], a DL-based channel estimation process is presented, where the signal, along with pilot signals, is transmitted. The effects of the channel on the pilot signals are determined, and DL then estimates the channel attributes using the interpolated channel. In ref. [103], a deep neural network (DNN)-based approach for channel estimation and symbol detection in an OFDM system is proposed. With offline training using OFDM samples generated from different information sequences under distinct channel conditions, the obtained model is used to recover the transmitted information without estimating the channel characteristics.

The automotive sector represents another highly significant scenario that has the potential for extensive development with the advent of 6G technology. The expansions of IoT, edge computing, and mobile AI have enabled urban authorities to leverage the wealth of data gathered from connected and autonomous vehicles (CAVs). In ref. [104], the authors propose an intelligent hierarchical framework for road infrastructure maintenance that harnesses the advancements in 6G communication technologies, deep learning techniques, and mobile edge AI training methods. The developed framework meets the stringent requirements for training efficient ML applications designed for CAVs and effectively utilizes the expected proliferation of CAVs on future road networks.

Moreover, 6G—considered the next major transformative technology in the telecommunications industry—is attracting significant attention from academia and businesses. The global COVID-19 pandemic has accelerated the adoption of virtual meetings and live video interactions across various sectors, including healthcare, business, and education.

Federated learning (FL) holds promise as a training approach for achieving pervasive intelligence in future 6G communication systems. However, implementing FL in 6G-enabled edge systems is challenging due to the high energy consumption during decentralized training and the limitations of battery-powered, resource-constrained mobile devices. The accumulation of intensive computations and communication costs from local updates during numerous global rounds has created an energy bottleneck, which is exacerbated when dealing with non-identical and independently distributed data. To address these challenges, FedRelay [105] represents a versatile multi-flow relay learning framework that performs local updates relay-by-relay in the training flow through model propagation, introducing a decentralized relay selection protocol that leverages the diversity of cooperative communication networks.

A CNN-based channel estimation framework for massive MIMO systems is proposed in ref. [106]; it utilizes one-dimensional convolution to process the input data. Ref. [107] presents an assisted IoT-oriented MIMO wireless network system optimized for 6G. This system aims to improve the bit error rate (BER) and capacity to overcome key challenges related to wireless communication.

### 7.3. Blockchain-Based Solutions

Existing traditional UAV communication methods are insufficient at addressing the high mobility and dynamic characteristics of UAVs, particularly in hostile environments. Therefore, there is a pressing need for an efficient and secure UAV network. Motivated by these factors, ref. [108] presents a comprehensive survey on the architecture, requirements, and use cases of 6G technology in the context of UAV communication. A taxonomy of solutions is proposed based on the applications of UAV communication, and a security solution leveraging blockchain technology and 6G-enabled network connectivity is introduced. A case study illustrating a blockchain-enabled UAV communication system using 6G networks to enhance the security of Industry 4.0 applications is also outlined.

Leveraging blockchain technology for diverse applications in SCs presents significant challenges to the private sector, public sector, and government entities. Providing improved and optimal services to the citizens is complex. Blockchain technology offers a solution for implementing effective policies, fostering trust among policymakers, and reducing data storage costs while ensuring data security. Its decentralized nature provides enhanced security, enabling anyone to verify records. Trust plays a crucial role in financial transactions and other dealings, and blockchain facilitates the restoration and maintenance of trust through its transparent system, allowing people to verify and monitor policy processes. This concept holds particular importance in the realm of banking, which significantly impacts individuals’ lives. Furthermore, the advent of 6G will revolutionize remote communication through the utilization of AI [109]. In ref. [110], the authors investigate the integration of blockchain technology in 6G networks, enabling efficient monitoring and management of resource consumption and sharing. In addition, solutions associated with security and privacy implications in 6G networks are examined, providing valuable insights and directions for future studies on 6G security and privacy. Recognizing the potential paradigm shift and security benefits brought by blockchain, transitioning from a traditional centralized model to a more robust and resilient decentralized model, the authors of [111] propose a multi-tier integrated architecture that combines blockchain and edge computing for 5G and future generations, aimed at addressing security challenges faced by resource-constrained edge devices. Moreover, robust security and privacy measures are necessary for various IoT-enabled industrial applications. The arrival of blockchain technology has revolutionized information sharing by establishing trust on a secure and distributed platform, eliminating the need for third-party authorities [112]. Notably, blockchain technology (BCT) has garnered significant attention due to its ability to address decentralization, transparency, spectrum resource scarcity, privacy, security, interoperability, and confidentiality, as well as its potential in emerging domains, such as industrial IoT (IIoT) and Industry 5.0 applications. The disparity between the requirements for data-intensive disruptive IoT applications and the capabilities of 5G networks has generated a demand for decentralized BCT-based architecture in 6G networks. Ref. [113] presents an extensive survey that explores the integration of blockchain in 6G mobile networks, IoT technologies, and smart industries.

### 7.4. Quantum Communication

With the world experiencing rapid technological advancements, there has been a substantial increase in the demand for communication that is ultra-reliable, fast, low-power, and secure. Consequently, researchers have taken a keen interest in the emerging field of QC due to its potential to solve complex computations in a robust and efficient manner. It is anticipated that QC can serve as a critical enabler and a powerful catalyst in significantly reducing computing complexities while enhancing the security of sixth-generation (6G) and future communication systems. The study presented in ref. [114] delves into the fundamentals of QC, with the evolution of quantum communication spanning various technologies and applications; the authors specifically focus on quantum key distribution as a promising application for quantum security. Additionally, the authors investigate various parameters and important techniques to optimize the performance of 6G communication in terms of security, computing, and communication efficiency. Potential challenges that QC and quantum communication may encounter in the context of 6G are highlighted, along with future directions for exploration. In ref. [115], the authors focus on exploring the security risks associated with the new 5G paradigm and propose solutions to address them. Specifically, we delve into the role of quantum key distribution as a means to enhance security in the context of 6G. Moreover, the work presented in ref. [116] introduces a novel approach that combines quantum cryptography and CNN for secure communication in SCs. The proposed approach involves the utilization of quantum key distribution (QKD) for the secure key exchange between communicating parties. The key generated through QKD is then employed to encrypt the data using a CNN. This CNN-based encryption adds an extra layer of security to the transmitted data within SCs.

Among the enabling technologies and solutions discussed in ref. [117], we cannot fail to mention quantum optical switching and computing, THz-to-optical conversions, advanced meta-materials for smart radio-optical programmable environments, and AI. This work presents a future application scenario called quantum optical twin, which utilizes the aforementioned quantum optical communication technologies to provide services such as ultra-massive scale communications for connected spaces and ambient intelligence, holographic telepresence, tactile internet, new paradigms of brain–computer interactions, and innovative forms of communication.

### 7.5. UAVs and Drone-Based Solutions

Another area that is gaining momentum is that of drones and their varied applications for use. The advent of consumer drones, flying ad hoc networks, low-latency 5G, and advancements beyond 5G have significantly accelerated progress in this field. The authors of [118] describe a continuous actor–critic deep Q-learning (ACDQL) strategy to solve the location optimization problem of UAV-BSs in the presence of mobile endpoints, extending the action space of the reinforcement learning (RL) algorithm from discrete to continuous. In detail, a scheme for the dynamic positioning of a UAV-BS in the case of user mobility is proposed, overcoming previous works presented in the literature, which considered fixed locations for the ground endpoints. The proposed reward function aims to keep the UAV-BS inside the boundaries of the area of interest, and it also intends to maximize the users’ sum data rate.

In ref. [119], the authors propose implementing the Consumer Internet of Drone Things (CIoDT) framework, which facilitates the transfer of emergency messages between smart grid systems and power sources, and ensures reliable network connectivity during a disaster. To achieve this, a realistic mobility model was developed, and an edge-enabled opportunistic MQTT message transfer mechanism was implemented. Additionally, a dedicated network slice was devised to assess routing performance, achieving a message delivery probability of about 0.99 in the quality of service level 2 (QoS2) and an end-to-end latency of 1.19 s in the quality of service level 1 (QoS1). In ref. [120], the authors present a continuous Hopfield neural network (CHNN) algorithm for the optimal link-state protocol (OLSR) routing, which is able to reduce breakage and provide efficient routing services in multiple types of UAVs, which form flying ad hoc networks (FANETs). This is obtained using self-assembled networks to collaborate on complex task-application scenarios, such as SCs. The proposed protocol outperforms the original OLSR protocol in key performance metrics, such as packet delivery rate, control overhead, throughput, and end-to-end delay.

Distributed hash table (DHT)-based routing protocols were initially proposed as valuable solutions in high-mobility cases characterized by frequent changes in the network topology. A novel three-dimensional logical cluster-based DHT routing protocol for mobile ad hoc networks (MANETs) that use logical clustering, as well as an effective replication strategy to reduce the routing overhead and lookup latency introduced by the above problems are proposed in ref. [133]. Ref. [134] proposed a subscriber data management scheme based on the combination of distributed ledger technology (DLT) and DHT technologies for 6G mobile communication systems. Traditional mobile communication systems are structured to serve numerous subscribers simultaneously, following a network-centric design approach. In contrast, this study explores the user-centric design approach, where systems are user-defined, user-configurable, and user-controllable. This approach allows for personalized network services tailored to each user. In ref. [135], the authors introduce a new user-centric network (UCN) architecture that outlines core design principles, relevant network components, and procedures. The UCN architecture employs DLT and DHT for distributed implementation, fostering customization, autonomous data control, and privacy protection.

With their affordability and the ability to integrate transmitters, cameras, and sensors, UAVs have the potential to serve as flying IoT devices, seamlessly connecting with their surroundings and offering enhanced mobility within the network. In ref. [121], an overview of the advantageous applications of UAVs in SCs, particularly in the realm of ITS, while emphasizing the key challenges that may arise, is presented. Additionally, the integration of various AI techniques enhances data delivery through global communication, thereby improving operational performance. Despite being in the early stages, UAV-enabled IoE applications hold immense potential for enhancing 6G networks. This paper investigates the recent advancements in methods and mechanisms that enable UAVs to support IoE. It explores the current trends in IoT convergence toward smarter IoE, while also addressing challenges and risks associated with the Internet of Everything [122].

### 7.6. Vehicular and Mobile Solutions

As presented in the previous section, MEC could solve challenges where transmitting data over long distances from end devices or edge servers to the cloud incurs great latency and security risks. In ref. [123], the authors analyze how MEC should improve applications and services, even in an SC environment. In contrast to cloud computing, edge computing provides a distributed computing platform for services, computation, and storing. MEC may decrease latency to lower levels and improve storage capability in devices with minimal processing capabilities, thanks to the growing use of smart systems. The incompatibility of various technology in cities is one of the most serious problems. In a scenario where MEC is used, a high number of connected devices might negatively impact the network speed, but they can be useful in identifying abnormal occurrences thanks to user-generated material and the right algorithms.

On the path to 6G, the advancing digitalization of buildings and SCs is becoming increasingly impactful [124]. To economically integrate renewable energy into buildings and SCs, the concept of energy storage and supply based on energy management must be emphasized. There has been a shift in political views; they now recognize the importance of energy efficiency in buildings given their considerable share (35%) of greenhouse gas emissions from final energy consumption. The authors highlight the potential of future mobile communication standards, such as 5G beyond and 6G, in providing specialized solutions for building digitization in edge clouds. The advent of 6G will revolutionize how we communicate and manage billions of interconnected devices in our digital future, spanning from macro- to micro- to nanoscales. Beyond providing lightning-fast connectivity, 6G has the potential to significantly enhance healthcare systems, transportation, logistics, safety measures, privacy, and more. It will enable the rapid processing, storage, and visualization of massive amounts of data, exploring the impact of 5G and 6G technologies on the advancement of SCs that are characterized by their intellect and perceptiveness [136].

The utilization of a microcell structure, employing carrier frequencies significantly higher than current 5G cellular networks, will present substantial challenges to the advancement of cellular communication in future generations. Specifically, the reliable operation of communication systems supporting critical services can be greatly impacted by factors like variations in vehicle velocity, rain-induced attenuation, and depolarization. The inclusion of eCall emergency systems—as a mandatory requirement in motor vehicles sold throughout the European Union since 2018—poses significant challenges to the development of 6G in emergency management, particularly due to the high occurrence of road accidents during heavy rainfall. To address the pressing need for assessing the reliability of 6G-based emergency management systems in SCs, in ref. [125], the authors thoroughly examine the technical aspects of designing and implementing vehicle-to-infrastructure (V2I) communication systems. The aim is to optimize reliability in an SC environment, considering the coexistence of both 5G and 6G network backbones. The main objectives outlined in ref. [126] focus on efficiently monitoring the intricate road environment in SC transportation using cutting-edge 6G digital twins (DTs). The aim is to perceive the complex road conditions within SC traffic, with a specific focus on vehicular networks (VNs) as the research subject. The study explores the potential for multi-sensor collaboration and fusion technology within the network to meet the active control requirements of intelligent vehicles. A segmentation network called C-LNet, which combines LiDAR and camera data, is proposed. C-LNet utilizes a double encoder–single decoder structure, with separate encoders extracting image and LiDAR features. By synchronizing LiDAR point cloud data and image data in the sensor space, the heterogeneous data are effectively unified. Additionally, a multiscale feature fusion-based method is designed to handle multimodal information related to vehicle collaboration. In ref. [127], the development of SCs relies on key solutions, such as 5G, IoT, automotive advancements, energy systems, high-speed digital capabilities, AI, data analytics, satellite communication, optics, and cyber-security. The integration of these systems and resources allows for the merging of mobility, communications, energy, water management, monitoring/control, performance management, predictability, and forecasting. Moreover, 6G enables the rapid sensing, processing, control, and human experience of vast amounts of data. The work aims to analyze the advantages and requirements of 6G technology and its potential impact on the development of smart, intelligent, and advanced cities. The potential of 6G joint communication and sensing (JCAS) in driving innovations and SC services highlights the use of wireless networks and millimeter-wave frequencies for perceptive radio networks and channel-based sensing [128]. The proposed concept focuses on operating in the mm-Wave beam space and measuring inter-cell links to enable more accurate spatial sensory activity monitoring. A simulation-based case study on vehicle detection and classification demonstrates the feasibility of the 6G-driven concept. The enhancements offer new service potentials, including recognizing the trajectory of passing road users.

In current computing systems, like edge computing and cloud computing, many emerging applications and practical scenarios are either unavailable or only partially implemented. This limitation has sparked interest in developing a comprehensive computing paradigm in both academia and industry. However, there is a gap in the existing research, with limited studies on the systematic design and review of such a comprehensive computing paradigm. To bridge this gap, this study introduces a new concept called aerial computing, which combines aerial radio access networks and edge computing. In ref. [129], the authors propose a novel comprehensive computing architecture composed of low-altitude computing (LAC), high-altitude computing (HAC), and satellite computing platforms, along with conventional computing systems. Aerial computing offers several desirable attributes, including global computing service, improved mobility, increased scalability and availability, and simultaneity. The study also explores key technologies that enable aerial computing, such as energy refilling, edge computing, network software, frequency spectra, multi-access techniques, AI, and big data. It discusses various vertical domain applications, such as SCs, smart vehicles, smart factories, and smart grids, which can benefit from aerial computing.

Among the different sectors, emergency services play a crucial role in the realization of future SCs. In this regard, firefighting stands out as a vital component for ensuring security and safety. However, effective firefighting necessitates ultra-reliable and low-latency communications, enabling firefighters to receive real-time guidance through the utilization of distributed sensors, robots, and other technologies. In this context, in ref. [130], the research focuses on the application of edge micro-data centers (EMDCs) to enhance fire prediction and management. A novel three-stage architecture is proposed. The initial stage involves predicting and classifying the occurrence of fires based on sensor data available at the EMDC. The second stage focuses on confirming the fire occurrence using a CNN classification model. Once the fire occurrence has been confirmed, the final stage involves notifying the tenants and streaming a 360-degree monitoring video to the nearby fire station after processing at the EMDC.

### 7.7. Augmented and Virtual Reality

The use of AR and VR technologies in the context of vehicular technologies in SCs will also benefit from the new features of 6G networks. In the SC context, vehicles are expected to use local (V2V) and global V2X communications to allow for safe, more efficient, and more comfortable driving, since the vehicle will be able to recognize dangerous situations preemptively, even if these are out of visual range due to a bend, or with other vehicles ahead [131]. For example, in ref. [132], the following situation is presented: Vehicles are moving—one behind the other—when suddenly, a pedestrian crosses the road in front of the first car. The first vehicle camera detects the situation and shares the image of the pedestrian with the vehicle behind it. The vehicle processes the information and shows a visual alert on the windshield along with the image of the pedestrian in AR. This use case requires high reliability, availability, low latency, and a high data rate, all of which can be assured by employing 6G communication techniques [131].

## 8. Real Implementation of Smart Cities

While there is presently no universally established set of guidelines for delineating a smart city, it is generally acknowledged that, globally, there are over 140 intelligent urban centers at the moment; this number is swiftly growing [137].

According to the latest smart city (SC) index, London has consistently risen in recent years and it currently holds the leading position [137]. London has introduced numerous progressive initiatives for smart cities, exemplified by the Connect London program, which oversees the development of an advanced 5G network and comprehensive fiber-optic coverage in the city [138]. Additionally, London has equipped its iconic lampposts with an array of sensors and electric vehicle charging points. Given the city’s substantial investments in artificial intelligence (AI), Internet of Things (IoT), and emerging 6G technology, these innovative solutions are expected to advance even further.

Barcelona is another prime example of a smart city. It has implemented a vast grid of LED lampposts outfitted with sensors. These sensors oversee traffic flow, air quality, pedestrian movement, and noise levels, and possess the capability to dim or deactivate lights as necessary. In addition, the city has introduced intelligent waste bins outfitted with vacuums that draw trash underground, reducing unpleasant odors, and cutting down the frequency of collection truck journeys. Oslo showcases another instance of smart sensor deployment. The Oslo Smart Street Lighting initiative encompasses the entire city and aims to enhance the efficiency of the street lighting network. Oslo has consolidated its street-lighting into a unified network that is accessible remotely through internet-based applications. This system permits managing and monitoring lighting levels, and adjusting light intensity based on seasonal variations and specific requirements, thereby further optimizing energy consumption across the city. The incorporation of 20,000 intelligent streetlights in Oslo has resulted in an impressive overall energy reduction of almost 70% [139].

Singapore holds a prominent position among the leading smart cities in Asia. With the initiation of its Smart Nation initiative in 2014, Singapore has introduced an extensive array of intelligent technologies. Notably, contactless payment technology has been widely integrated into streamline movements and payment processes for the approximately 7.5 million individuals using public transportation in Singapore. In response to the challenges posed by an aging population, a digital health system was implemented, which not only normalized video consultations but also introduced wearable Internet of Things (IoT) devices for patient monitoring. This multifaceted approach serves to alleviate the pressures associated with an aging demographic [139].

Seoul is notably regarded as the most advanced city in terms of harnessing big data. By gathering and analyzing urban patterns, encompassing factors like traffic flow, speed, and air quality through an array of sensors and closed-circuit television installations distributed throughout the city, Seoul has laid a robust foundation for the development of intelligent infrastructure and services. A specific focus has been directed toward addressing the needs of the city’s aging population. An initiative promoting safety for senior citizens living alone was introduced. This initiative involves the utilization of environmental sensors. These sensors detect anomalies, such as prolonged inactivity or unusual temperature, humidity, or lighting conditions. In such cases, the system immediately notifies relevant case workers and emergency services. Moreover, Seoul is exploring the idea of using its data platform to create an AI-powered detective system that can identify potential crime patterns [140]. Additionally, the city is among the pioneers in adopting 5G technology for enhancing mobility and transportation systems [139].

Finally, New York serves as another testament to the adoption of advanced technologies for SCs. Hundreds of smart sensors and technologies have been tested and placed throughout the different districts in New York City as part of its SC pilot program in 2020 [141]. This program gathers data to help manage services (like waste management and collection) more efficiently. New York replaced their old phone booths with smart hubs equipped with contactless technology, Wi-Fi capabilities, as well as online charging stations [140].

## 9. Challenges and Conclusions

SCs and 6G are technologies that will transform the way we live and work in the near future. However, combining these technologies leads to a series of challenges that have to be addressed. The challenges for SCs include interoperability, data privacy and security, digital divide, and implementation challenges. Interoperability means that SCs rely on a wide range of connected devices and systems; ensuring that these systems can communicate with one another is a major challenge. Regarding data privacy and security, we can see that SCs generate vast amounts of data, much of which are sensitive and personal. To maintain public trust, it is crucial to ensure that these data are stored and processed securely. In agreement with the digital divide, SC technology is expensive and not accessible to everyone, leading to a digital divide between those who can afford it and those who cannot. Lastly, implementing SC technology requires a significant investment in infrastructure and resources, which can be challenging for some cities.

The challenges of 6G cover different areas, such as technical hurdles, spectrum availability, costs, security, and regulatory challenges. In fact, 6G will require new infrastructure and technology, which may be difficult to develop and deploy. Moreover, 6G will require a significant amount of spectrum availability, which may not be the case in some regions; developing and deploying 6G will be expensive, and there may be concerns about the cost of accessing 6G services. As with any new technology, there may be security concerns with 6G that must be addressed to ensure the safety and privacy of users. Finally, the development and deployment of 6G will require cooperation between governments and private industries, and there may be regulatory hurdles that need to be overcome. Achieving global coverage with 6G will also require international cooperation and coordination. Frequency allocation and network standards will need to be agreed upon and implemented globally, which could be challenging given the competing interests of different countries and network providers. One of the main challenges of global coverage with 6G is the need for a significant infrastructure upgrade. The 6G networks will require many more base stations and transceivers than the current 5G networks to provide adequate coverage, which will require significant investments by network providers and governments. Another challenge of global 6G coverage is the allocation of frequency bands. While 6G networks will likely use higher frequency bands than 5G networks, these frequencies have shorter wavelengths, which means they are more susceptible to attenuation by obstacles such as buildings and trees. This could require additional infrastructure investments to ensure that 6G signals can penetrate obstacles and provide adequate coverage.

SCs rely heavily on advanced telecommunication technologies to support the development of innovative solutions for public services, transportation, energy management, and other applications. Considering this, 6G networks are expected to play a crucial role in enabling the next generation of SCs by providing faster data transfer speeds, lower latency, and increased connectivity. Some potential use cases for 6G in SCs include enhanced mobility, remote healthcare, smart energy management, and public safety. Moreover, 6G networks can support real-time data transfers, enabling advanced driver-assistance systems and autonomous vehicles to communicate seamlessly with one another and SC infrastructure. In addition, 6G networks can facilitate remote healthcare services, including telemedicine, remote patient monitoring, and virtual consultations. One of the aspects that is becoming more prominent is represented by smart energy management, where 6G networks can help power smart grids by enabling the real-time monitoring and control of energy consumption, facilitating the integration of renewable energy sources, and improving energy efficiency. Finally, public safety in SCs can be improved by employing 6G networks to support advanced surveillance systems, emergency response services, and disaster management, by providing fast and reliable communication channels. Overall, 6G networks have the potential to transform how cities function by providing faster, more reliable, and more efficient connectivity, supporting a wide range of SC applications.

This survey tackles all the aforementioned challenges and topics and provides an extensive overview of the published scientific work for each particular topic. Specifically, we concentrated on papers examining SCs in the context of 6G, building on the foundation set by the existing 5G standard and its inherent limitations. We extended the survey beyond the mere delineation of the 6G standard, delving into the potential applications that this emerging standard promises to bring to the SC realm.

## Figures and Tables

**Table 1:** The 5G features and benefits.

5G Features	5G Benefits
Fastest response time	Wide bidirectional bandwidth
High capacity	Unification of radio communications
Connectivity of up to 25 Mbps	Easily integrated with previous generations
Bidirectional, wide bandwidth	Ability to provide uniform and uninterrupted connectivity
Supports private virtual networks	

**Table 2:** Comparison between 5G and 6G technologies.

5G	6G
Sub-6GHz band crowded	Ultra-fast connection (THz)
Limited capacity for new type of communications	Fully immersive extended reality (XR) experience and high definition holographic communication
Cellular communications	Cell-free massive MIMO
Infrastructure	Ubiquitous connectivity
Security problems	Quantum communication and Blockchain
Low latency	High reliability and ultra-low latency
Existing algorithms for modeling channel information	Deep learning as solution for improvement

**Table 3:** The 6G enabling technologies for SCs.

Technology	Short Description
Terahertz Communications (THz)	THz waves refer to frequencies 0.1–10 THz with the corresponding wavelengths in the 0.03–3 mm range
Artificial Intelligence (AI)	AI for automatization enabling the transformation from cognitive radio to intelligent radio also improving the transport of real-time data
Massive MIMO (M-MIMO)	5G massive MIMO technology will converge toward IRS in 6G wireless systems that is massive MIMO 2.0
Blockchain (BC)	Perfect technology to manage massive data in future communication systems with improved privacy and security, scalability, interoperability, and reliability
Quantum communication (QC)	Emerging paradigm of quantum computing able to improve its security by using a quantum key based on the quantum no-cloning theorem and uncertainty principle
Cell-free communications(CFC)	Users will be able to move from a network to another choosing and selecting the best one automatically solving issues related to handover failures delays and similar side effects
UAVs	BSs on board to provide cellular connectivity, with specific features as easy deployment of strong line-of-sight links, and degrees of freedom with controlled mobility
Mobile edge computing (MEC)	Able to solve challenges where the long-distance transmission of data from end devices or edge servers to the cloud incurs great latency and security risks
Optical wireless (OW)	These communication technologies will provide very high data rates and low latencies
Augmented/virtual reality (AR/VR)	These technologies will enable new immersive applications and services

## Abbreviations

**Acronym**	**Description**
6G	Sixth-Generation
5G	Fifth-Generation
IoT	Internet of Things
SC	Smart City
AI	Artificial Intelligence
IaaS	Infrastructure as a Service
PaaS	Platform as a Service
M2M	Machine to Machine
RAT	Radio Access Technology
LTE	Long-Term Evolution
QoS	Quality of Service
ARMANS	Adaptive Real-time Multi-user Access Network Selection
E-ARMANS	Energy-aware ARMANS
TYDER	Network Traffic Type-based Differentiated Reputation
M-IoT	Multimedia-based IoT
ML	Machine Learning
MCS	Modulation and Coding Scheme
ALOW	Additive Logarithmic Weighting
GATH	Game Theory
ITU	International Telecommunication Union
ITS	Intelligent Transportation Systems
ETA	Estimated Time of Arrival
AQI	Air Quality Index
SBS-SVO	Smart Bus Stop–Social Virtual Object
MISSION	Mobility Services User Virtualization
SIoT	Social IoT
LPWANs	Low-Power Wide Area Networks
AR	Augmented Reality
VR	Virtual Reality
e2McH	Multi-Hop Communication Solution
QoE	Quality of Experience
RAN	Radio Access Network
SDN	Software-Defined Networking
NFV	Network Function Virtualization
ITU	International Telecommunication Union
IMT	International Mobile Telecommunications
FBMC	Filter Bank-based Multi-Carrier
UFMC	Universal-Filtered Multi-Carrier
NOMA	Non-Orthogonal Multiple Access
SCMA	Sparse Code Multiple Access
MIMO	Multiple-Input Multiple-Output
CF-mMIMO	Cell-Free massive MIMO
ESS	Energy Self-Sustainability
UAD	User Activity Detection
D2D	Device to Device
EPS	Evolved Packet System
GSMA	Groupe Speciale Mobile Association
ICT	Information and Communications Technology
OPEX	Operator Experience
UDN	Ultra-dense Network
CRAT	Context-aware Radio Access Technology
AHP	Analytical Hierarchical Process
TOPOSIS	Technique for Order of Preference by Similarity to Ideal Solution
OFDM	Orthogonal Frequency-Division Multiplexing
CRAN	Cloud RAN
LB	Load Balance
IAB	Industry Advisory Board
WRC	World Radiocommunication Conference
3CLS	Control Localization and Sensing
BCI	Brain–Computer Interface
LiDAR	Light Detection and Ranging
SLAM	Simultaneous Localization and Mapping
UAV	Unmanned Aerial Vehicle
BS	Base Station
MEC	Mobile Edge Computing
MEEC	Mobility-Enhanced Edge Computing
V2X	Vehicle-to-Everything
FSO	Free-Space Optical
BC	Blockchain
QC	Quantum Communication
CFC	Cell Free Communication
OW	Optical Wireless
DL	Deep Learning
IRS	Intelligent Reflecting Surface
RIS	Reconfigurable Intelligent Surface
mmWave	Millimeter Wave
CNNs	Convolutional Neural Networks
RNNs	Recurrent Neural Networks
DNN	Deep Neural Network
CAV	Connected and Autonomous Vehicle
BCT	BC Technology
QKD	Quantum Key Distribution
ACDQL	Actor–Critic Deep Q-Learning
RL	Reinforcement Learning
CIoDT	Consumer Internet of Drone Things
CHNN	Continuous Hopfield Neural Network
OLSR	Optimal Link-State Protocol
FANETs	Flying ad hoc Networks
V2I	Vehicle-to-Infrastructure
DT	Digital Twin
VN	Vehicular Network
LAC	Low-Altitude Computing
HAC	High-Altitude Computing
EMDC	Edge Micro Data Center
V2V	Vehicle-to-Vehicle
